# Sexual activity, vaginal symptoms, maternal perineal hygiene behavior, and constipation on ano-vaginal colonization of group B streptococcus in near term pregnancy

**DOI:** 10.1186/s12884-024-06616-7

**Published:** 2024-07-04

**Authors:** Ruziyati Esmaon, Boon Kiong Lim, Farah Gan, Mukhri Hamdan, Peng Chiong Tan

**Affiliations:** Department of Obstetrics and Gynecology, Faculty of Medicine, University Malaya, Jalan Profesor Diraja Ungku Aziz, Kuala Lumpur, 50603 Malaysia

**Keywords:** Group B streptococcus, Streptococcus agalactiae, Risk factor, Predictor, Sexual intercourse, Antibiotic, Recent coitus, panty liner use, and antenatal vaginal irritation are independent associates of ano-vaginal Group B Streptococcus colonization in late pregnancy

## Abstract

**Background:**

Maternal Group B Streptococcus (GBS) colonization is influenced by many factors but results are inconsistent. Consideration of antenatal risk factors may help inform decision making on GBS microbiological culture screening where universal screening is not standard of care. We sought to identify independent predictors of GBS colonization at 34–37 weeks gestation incorporating vaginal symptoms, perineal hygiene measures, sexual activity, and a potential novel factor, constipation.

**Methods:**

In this prospective cross-sectional study, 573 women at 34–37 weeks gestation had an ano-vaginal swab taken and sent for selective culture for GBS. Women were asked about vaginal bleeding, discharge, irritation and candidiasis, antibiotic use during pregnancy, ano-vaginal hygiene practices such as douching and perineal cleansing after toileting, sexual intercourse related activities, and a potential novel factor for GBS carriage, constipation. Maternal basic demographics and obstetric-related characteristics were also collected. Bivariate analyses were performed to identify associates of GBS colonization. All variables with *p* < 0.05 found on bivariate analysis were then included into a model for multivariable binary logistic regression analysis to identify independent risk factors for GBS colonization.

**Results:**

GBS colonization was found in 235/573 (41.0%) of participants. Twenty six independent variables were considered for bivariate analysis. Eight were found to have *p* < 0.05. Following adjusted analysis, six independent predictors of GBS colonization were identified: ethnicity, previous neonatal GBS prophylaxis, antenatal vaginal irritation, antibiotic use, recent panty liner use, and frequency of sexual intercourse. Vaginal discharge and perineal cleansing were not associated after adjustment. Recent douching and constipation were not associated on bivariate analysis.

**Conclusion:**

The identification of independent predictors of GBS colonization in late pregnancy may inform the woman and care provider in their shared decision making for microbiological screening at 35–38 weeks gestation in locations where universal GBS screening is not standard of care.

**Ethics oversight:**

This study was approved by the Medical Ethics Committee of University Malaya Medical Centre (UMMC) on August 9, 2022, reference number 2022328-11120.

**Supplementary Information:**

The online version contains supplementary material available at 10.1186/s12884-024-06616-7.

## Introduction

Approximately 19·7 million pregnant women were estimated to have rectovaginal colonization with Group B streptococcus (GBS) in 2020 [1] with at least 409 thousand (95% confidence interval: 144–573 thousand) maternal/ fetal/ infant cases and 147 thousand (uncertainty range, 47–273 thousand) stillbirths and infant deaths annually [2]. The primary risk factor for neonatal GBS early-onset disease (EOD) is GBS colonization of the maternal genitourinary and gastrointestinal tracts. Approximately 50% of women who are colonized with GBS will transmit the bacteria to their newborns. In the absence of intrapartum antibiotic prophylaxis, 1–2% of those newborns will develop GBS EOD [3]. GBS EOD has 5.2% mortality and 7.4% disability rate [4].

Asymptomatic colonization rates of pregnant women with GBS in the vagina or rectum varies between 6.5% [5] and 43.6% [6]. Maternal GBS colonization is influenced by age [7, 8], parity [9, 10], ethnicity [10–12], body mass index [8, 10, 13–15], GBS colonization in previous pregnancy [16–18], vulvitis [11], presence of sexually transmitted diseases [11, 19, 20], sexual behavior [21, 22], tobacco use [11, 23, 24], antibiotic exposure in pregnancy [25, 26], diabetes [27–34], and healthcare worker occupation [35] but results were inconsistent.

Intrapartum antibiotic prophylaxis is recommended in the presence of previous neonate with GBS disease, positive screening culture in the last 5 weeks, GBS bacteriuria in pregnancy, known GBS positive result in a previous pregnancy and intrapartum preterm birth, membrane rupture > 18 h, and maternal fever ≥ 38 ^0^C [3].

The American College of Obstetricians and Gynecologists recommends universal GBS screening between 36 + 0/7 and 37 + 6/7 weeks of gestation [3]. In contrast, the United Kingdom National Screening Committee recommended that routine screening using bacteriological culture or near-patient testing techniques should not be introduced into United Kingdom practice, citing very low GBS EOD rates and the large number of women that would be given intrapartum antibiotic prophylaxis with universal screening [4]. Universal GBS screening is not standard of care in our Malaysian practice.

Considering antenatal risk factors may help inform decision-making on GBS microbiological culture screening where universal screening is not the standard of care. We sought to identify independent risk factors of GBS colonization at 34–37 weeks gestation [4] incorporating vaginal symptoms and sexual activity, and also as potential novel factors, perineal hygiene measures and constipation.

## Materials and methods

This is a prospective cross-sectional study, approved by the Medical Ethics Committee of University Malaya Medical Centre (UMMC) on August 9, 2022, reference number 2022328-11120. This study was conducted in UMMC with the first participant recruited on August 17, 2022 and the last on February 7, 2023. Women who attended the antenatal clinic for routine care in UMMC, Kuala Lumpur, Malaysia, were assessed for eligibility to be recruited into the study.

Inclusion criteria were pregnant women age at least 18 years old, pregnant at 34–37 weeks, and live fetus. We excluded women with retroviral disease, active vaginal bleeding, and prelabor membrane rupture.

Eligible women were approached, provided with the patient information sheet, and had verbal enquiries answered by the recruiting care provider. Written informed consent was obtained from the participants.

After recruitment, participants had the anovaginal swab collection for GBS culture and completed a self-reported questionnaire developed for this study (Supplementary Material 1) that included questions on vaginal bleeding, discharge, irritation, candidiasis, and antibiotic use during pregnancy, ano-vaginal hygiene such as douching, perineal cleansing after toileting, and sexual intercourse related activities. Functional chronic constipation was identified using the Rome IV criterion [36].

Care providers would swab the lower part of the vagina without inserting a speculum. The same swab was then inserted through the anal sphincter (endoanal), rotated two or three times, and placed into the culture medium. A single swab was used [4]. The culture medium used in this study is the non-nutritive transport medium (Amies media with or without charcoal) and the samples were sent to the UMMC laboratory on the same day for GBS selective culture. Women were informed of culture results and those with positive cultures for GBS were given intrapartum antibiotic prophylaxis.

Data for the impact size of our novel risk factors on GBS colonization is unknown. We made the assumption odds ratio was a generic 1.75 over the negative. Using the Chi-Square test (case-control) with alpha 0.05, beta 0.2, and baseline GBS carriage at 20%, assuming 1 to 1 ratio in dichotomization within the independent novel covariables and odds ratio of 1.75, 271 participants were needed in each half of the groups i.e., 542 in total.

Taking a baseline GBS carriage rate of 20%, the event rate is 0.20. Considering a 10 variables multivariable logistic regression analysis, utilizing the rule of at least 10 cases/events, 100 GBS carriers are needed which should be found in 500 participants. Hence, we plan to recruit at least 550 participants to cover both calculations.

SPSS statistical software (Version 26, IBM, SPSS Statistics) was used. Descriptive statistics were performed. For crude bivariate analyses, the Student t-test was used to analyze means with normally distributed data, the Mann-Whitney U test for non-normally distributed data or ordinal data and the Chi-square test (Fisher exact test used if cell size < 5 encountered in ≥ 20% of cells) for categorical data comparing women classified as GBS positive and GBS negative for ano-vaginal colonization. Variables with *p* < 0.05 on crude bivariate analysis were incorporated into the model for adjusted analysis (multivariable binary logistic regression) to identify independent risk factors of GBS colonization. 2-sided *p* < 0.05 is taken as the level of significance.

## Results

**Fig. 1:**
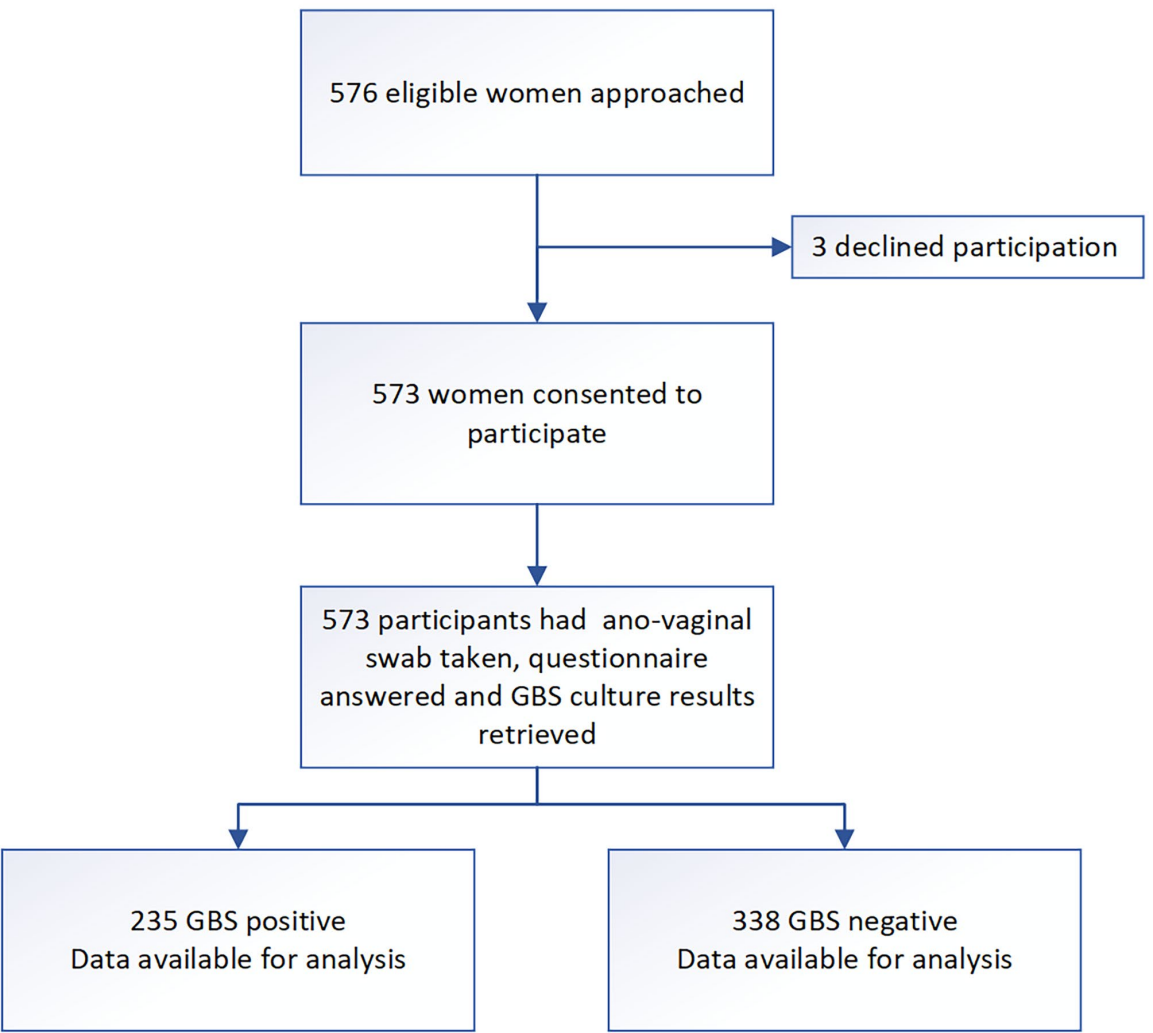
Recruitment flow chart into a prospective cross-sectional study on ano-vaginal Group B streptococcus colonization at 34–37 weeks gestation

Figure 1 depicts the recruitment flowchart of participants through the study. The first participant was recruited on August 17, 2022, and the last on February 7, 2023. Of 576 eligible women approached to participate, three declined. Five hundred and seventy-three (573) women provided informed written consent and were recruited into the study.

Table 1 shows the 26 selected factors for the entire study population, and then dichotomized and analyzed according to GBS positive and negative status. The *p*-value from the bivariate analysis of these factors against GBS colonization status were displayed. The bivariate analysis used the appropriate statistical test for the type of data. There were eight variables with *p* < 0.05 after bivariate analysis; ethnicity, previous neonatal GBS prophylaxis, vaginal discharge in pregnancy, vaginal irritation in pregnancy, antibiotic use in pregnancy (any indication), perineal cleanser after toileting (urination and bowel movement), panty liner use in the preceding two weeks, and sexual intercourse in the past two weeks.

**Table 1:** Characteristics of all women in the study and then dichotomized into those who were Group B Streptococcus (GBS) positive or GBS negative

Characteristics	All women (*n* = 573)	GBS positive (*n* = 235)	GBS negative (*n* = 338)	*P* value
**Maternal demographics**				
Age (years)	31.3[28.2–34.7]	30.9[28.0-34.8]	31.1[28.5–34.7]	0.608
BMI (kg/m^2^)	28.0[25.2–31.2]	28.3[25.2–31.2]	27.8[25.2–31.2]	0.635
Ethnicity				< 0.001
Malay	401 (70.0%)	188 (80.0%)	213 (63.0%)	
Non-Malay		47 (20.0%)	125 (37.0%)	
Chinese	112 (19.5%)	27 (11.5%)	85 (25.1%)	
Indian	51 (8.9%)	18 (7.7%)	33 (9.8%)	
Others	9 (1.6%)	2 (0.9%)	7 (2.1%)	
Occupation				
Healthcare providers	119 (20.8%)	58 (24.7%)	61 (18.0%)	0.054
Alcohol in pregnancy	2 (0.3%)	1 (0.4%)	1 (0.3%)	> 0.99^1^
Cigarette smoking in pregnancy	0 (0)	0 (0)		^2^
Sexually transmitted disease (lifetime)	5 (0.9%)	1 (0.4%)	4 (1.2%)	0.413^1^
Abnormal cervical smear (lifetime)	8 (1.4%)	3 (1.3%)	5 (1.5%)	> 0.99^1^
**Obstetric characteristics**				
Gestational age	36.0[35.1–36.6]	36.0[35.1–36.6]	35.9[35.0-36.6]	0.457
Nulliparous	258 (45.0%)	98 (41.7%)	160 (47.3%)	0.182
Previous miscarriage^3^	106 (18.5%)	46 (19.6%)	60 (17.8%)	0.580
Diabetes in pregnancy	133 (23.2%)	54 (23.0%)	79 (23.4%)	0.912
Previous preterm delivery^4^	29 (5.1%)	13 (5.5%)	16 (4.7)	0.668
Previous neonatal GBS prophylaxis	23 (4.0%)	15 (6.4%)	8 (2.4%)	0.016
Vaginal bleeding in pregnancy	45 (7.9%)	20 (8.5%)	25 (7.4%)	0.626
Vaginal discharge in pregnancy	354 (61.8%)	163 (69.4%)	191 (56.5%)	0.002
Vaginal irritation in pregnancy	139 (24.3%)	84 (35.7%)	55 (16.3%)	< 0.001
Vaginal candidiasis in pregnancy	52 (9.1%)	26 (11.1%)	26 (7.7%)	0.167
Antibiotic use in pregnancy	130 (22.7%)	42 (17.9%)	88 (26.0%)	0.022
**Maternal perineal hygiene**				
Panty liner usage in the past 2 weeks	356 (62.1%)	171 (72.8%)	185 (54.7%)	< 0.001
Douching in the past 2 weeks				0.911
Daily	41 (7.2%)	18 (7.7%)	23 (6.8%)	
Occasionally	50 (8.7%)	21 (8.9%)	29 (8.6%)	
None	482 (84.1%)	196 (83.4%)	286 (84.6%)	
Perineal cleanser after bowel motion or urination				0.021^1^
Mainly water	516 (90.1%)	220 (93.6%)	296 (87.6%)	
Mainly toilet paper	55 (9.6%)	14 (6.0%)	41 (12.1%)	
Mainly wet wipes	2 (0.3%)	1 (0.4%)	1 (0.3%)	
**Coital activity**				
Coital frequency in the last 2 weeks				< 0.001
Nil	191 (33.3%)	55 (23.4%)	136 (40.2%)	
< 1 time per week	263 (45.9%)	120 (51.1%)	143 (42.3%)	
1–3 times per week	109 (19.0%)	54 (23.0%)	55 (16.3%)	
> 3 times per week	10 (1.7%)	6 (2.6%)	4 (1.2%)	
Lubricant use during coitus	30 (5.2%)	16 (6.8%)	14 (4.1%)	0.159
Withdrawal during coitus	22 (3.8%)	7 (3.0%)	15 (4.4%)	0.371
**Constipation**: ROME IV score ≥ 2/6	107 (18.7%)	44 (18.7%)	63 (18.6%)	0.980

Data expressed as median[interquartile range] and number (%). Data was analyzed using Mann Whitney U test for non-normally distributed continuous data and the Chi-Square test for categorical data

^1^ Fisher exact test applied instead of Chi-Square test as ≥ 20% of cells has value < 5

^2^*p*-value not calculated as cells with a null value

^3^ Pregnancy loss < 22 weeks

^4^ Delivery < 37 weeks

Table 2 presents the eight variables with bivariate *p* < 0.05 incorporated into the model for multivariable binary logistic regression analysis. This was the a priori model proposed for the study. The crude relative risk (95% confidence interval) and *p* value after bivariate analysis are shown, as well as the adjusted odds ratio (AOR) and *p* value following adjustment. After adjustment, six variables were found to be independent risk factors of GBS colonization. In order of their AOR on positive GBS colonization status, from highest AOR, the six significant independent risk factors are previous neonatal GBS prophylaxis AOR 3.18, vaginal irritation in pregnancy AOR 2.69, increasing frequency of sexual intercourse in the preceding two weeks AOR 1.74–2.57, panty liner use in the past two weeks AOR 1.75, Malay ethnicity AOR 1.72, and any antibiotic use in pregnancy AOR 0.47.

**Table 2:** Bivariate and multivariable binary logistic regression analysis of women dichotomized according to ano-vaginal Group B streptococcus (GBS) colonization status^1^

Characteristics	Positive (*n* = 235)	Negative (*n* = 338)	RR (95% CI)	*P* value	AOR (95% CI)	Adjusted *P* value
**Maternal demographics**						
Ethnicity				< 0.001		
Malay	188 (80.0%)	213 (63.0%)	1.27 (1.14–1.41)		1.72 (1.06–2.81)	0.029
Non-malay	47 (20.0%)	125 (37.0%)			^2^	
**Obstetric characteristics**						
Vaginal discharge in pregnancy	163 (69.4%)	191 (56.5%)	1.22 (1.08–1.39)	0.002	1.33 (0.89–1.99)	0.163
Vaginal irritation in pregnancy	84 (35.7%)	55 (16.3%)	2.20 (1.63–2.96)	< 0.001	2.69 (1.75–4.14)	< 0.001
Antibiotic in pregnancy	42 (17.9%)	88 (26.0%)	0.68 (0.50–0.95)	0.022	0.47 (0.30–0.74)	< 0.001
Previous neonatal GBS prophylaxis	15 (6.4%)	8 (2.4%)	2.70 (1.16–6.26)	0.016	3.18 (1.24–8.14)	0.016
**Maternal hygiene**						
Panty liner use in last 2 weeks	171 (72.8%)	185 (54.7%)	1.33 (1.17–1.51)	< 0.001	1.75 (1.18–2.64)	0.006
Perineal cleanser after bowel motion or urination				0.021		0.941
Mainly water	220 (93.6%)	296 (87.6%)			^2^	
Mainly toilet paper	14 (6.0%)	41 (12.1%)			1.01 (0.48–21.3)	0.978
Mainly wet wipes	1 (0.4%)	1 (0.3%)			1.68 (0.09–31.1)	0.727
**Sexual intercourse**						
Coital frequency in last 2 weeks				< 0.001		0.025
Nil	55 (23.4%)	136 (40.2%)			^2^	
< 1 time per week	120 (51.1%)	143 (42.3%)			1.74 (1.12–2.72)	0.014
1–3 times per week	54 (23.0%)	55 (16.3%)			2.11 (1.24–3.62)	0.006
> 3 times per week	6 (2.6%)	4 (1.2%)			2.57 (0.85–10.13)	0.177

Data expressed as number (%). Bivariate using t test for comparison of mean and Chi Square test for categorical data. All the eight variables in this table were entered into the model for multivariable binary logistic regression analysis to identify independent predictors of ano-vaginal GBS colonization in late pregnancy

^1^Table 2 lists the eight variables whose bivariate analysis for ano-vaginal GBS colonization in late pregnancy status showed *p* value < 0.05 (see Table 1)

^2^Referent characteristic

## Discussion

The prevalence of ano-vaginal GBS colonization in late pregnancy in this study was 41.0%. This is at the upper end of the prevalence range reported in the literature. A study has reported a maternal GBS colonization rate as high as 43.6% [6], contemporary studies report a 37.3% at labor induction [37] and 41.3% rate in women with HIV which was not different from their controls [38].

In our study, after adjustment, the factor with the strongest association for GBS colonization was previous neonatal GBS prophylaxis. Maternal GBS colonization in previous pregnancy has been consistently identified as a risk factor in other studies [16–18]. Maternal GBS colonization is a common indication for neonatal GBS antibiotic prophylaxis [4].

Vaginal irritation and itchiness, but not vaginal discharge, was the factor with the next highest odds ratio for GBS colonization in this study. Symptomatic vaginitis has been described in two women heavily colonized with GBS [39]. GBS colonization has also been associated with vulvitis [11]. In non-pregnant women, vaginal GBS colonization is associated vaginal burning/pain but the association is not significant after adjustment for urinary tract, yeast, and herpes simplex virus 2 infections [40].

We found ‘any antibiotic use in pregnancy; to be protective of GBS colonization after adjustment. Our findings corroborate those of a previous study that found the use of antibiotics active against GBS to be associated with a decreased rate of vaginal GBS colonization, but only when the rectum is not also colonized with GBS [40]. In contrast, other studies find that antibiotic exposure in pregnancy is a risk factor for GBS colonization (in univariate analyses but not after adjustment) [26], and also that prenatal antibiotic treatment does not decrease group B streptococcus colonization at delivery [25].

Coitus in the preceding two weeks was also found to be an independent risk factor of GBS colonization with a positive frequency-related trend. In support, in non-pregnant women, recent sexual intercourse increases the risk of vaginal GBS colonization [40] and a doubling in sex acts significantly increased the incidence of some GBS types by 40–80% [21]. In contrast, it has also been reported that sexual behavior does not predict vaginal colonization by GBS [22].

Malay ethnicity in our study cohort was independently predictive of GBS colonization. GBS colonization has been associated with ethnicity or race in several studies [10–12]. Recent use of panty liner was also independently predictive of GBS colonization, whilst factors that could precipitate panty liner use such as vaginal discharge in pregnancy were not predictive after adjustment. Vaginal bleeding in pregnancy was not even significant in bivariate analysis in our study.

We have not found within our data as others have for maternal GBS colonization to be associated with age [7, 8], parity [9, 10], body mass index [8, 10, 13–15], tobacco use [11, 23, 24], and healthcare worker occupation [35]. Constipation in pregnancy did not contribute to GBS colonization in our findings.

### Research implication

Further research focused on building a prediction calculator based on risk factors for GBS colonization at the time of GBS screening is warranted to assist decision making in locations where universal screening remains controversial. Our findings of an association between any antibiotic exposure, the frequency-dependent effect of recent vaginal intercourse, and recent panty liner use should generate interest for further investigation as these factors are plausibly remediable if proven to be causative.

### Strengths and limitations

As to strength, our study achieved target sample size of 573 with 235 GBS cases identified, more than adequate for the eight variables in our model for multivariable binary logistic regression. We applied multivariable binary logistic regression to reduce confounding. As to limitations, events were self-reported and subject to recall bias though culture results were not known at questionnaire completion. Our data which was from a single center might reduce generalizability. Using the same swab to sample both sites (anus and vagina) is sanctioned by the Centers for Disease Control and Prevention (CDC) [28] and the American Society for Microbiology [41]. However, it is plausible that culture from a single swab may not be as sensitive for anovaginal colonization as cultures from separate anal and vaginal swabs.

## Conclusion

The identification of independent predictors of GBS colonization in late pregnancy may aid in informing the woman and care provider in their shared decision making for microbiological screening at 35–38 weeks gestation in locations where universal GBS screening is not standard of care. Remediable factors of the protective effect of antibiotic exposure in pregnancy and the deleterious effect of recent sexual intercourse and panty liner use warrant further evaluation, not least as potential interventions.

### Electronic supplementary material

Below is the link to the electronic supplementary material.

Supplementary Material 1

## Data Availability

The datasets used and analyzed during the current study are available from the corresponding author on reasonable request.
