# Supplementary material for: Sexual activity, vaginal symptoms, maternal perineal hygiene behavior, and constipation on ano-vaginal colonization of group B streptococcus in near term pregnancy
Source: BMC Pregnancy Childbirth. 2024 Jul 4;24:461. doi: 10.1186/s12884-024-06616-7 (PMC11225375; doi:10.1186/s12884-024-06616-7)
Supplement: Supplementary file 1 — Supplementary Material 1 [file 12884_2024_6616_MOESM1_ESM.pdf]

STUDY NUMBER	*Pt's Sticker

CASE REPORT FORM

Date of data collection : __ / __ / __ (dd/ mm/ yy)

Part I : Patient's Demographic Information

Patient characteristics :

D.O.B : ______

Gravida: _____ Para: _____ Abortion/Molar/Ectopic: ______

EDD : __ / __ / __ (dd/ mm/ yy)

Ethnicity: _________

Occupation: * Healthcare / Non Healthcare

Please state current :

Weight : _________ kg

Height : _________ cm

BMI : _________ kg/m2

Part II : Questionnaires :

*** Please encircle your answer / tick in the box of your selected answer.**

1- Do you use pantyliner in the past 2 weeks? * Yes / No

- If yes, how frequent are you using it ? ☐ Daily ☐ Occasionally

2- Do you have any vaginal discharge throughout this pregnancy ? * Yes/ No

3- Do you have any vaginal itchiness/ irritation ? * Yes/ No

4- Do you use any vaginal douching for the past 2 weeks? * Yes/ No

- If yes, how frequent are you using it ? ☐ Daily ☐ Occasionally

5- Have you ever had any alcohol consumption during this pregnancy? * Yes/ No

6- Do you have any tobacco during this pregnancy? * Yes/ No

7- Did you consume any antibiotic course during this pregnancy? * Yes/ No

8- How was your vaginal intercourse in the past 2 weeks?

- frequency : ☐ ≤ 1x/2weeks ☐ 1-3x/week ☐ >3x/week ☐ Nil

-any withdrawal during intercourse? * Yes / No

-any use of lubricant during intercourse? * Yes / No

8- Cleaning behaviour after bowel motion or passing urine :

☐regularly use water ☐regularly use toilet paper ☐ regularly use wet wipes

Part III: Underlying risk :

1- Underlying Diabetes Mellitus (DM/GDM) in this pregnancy : * Yes / No

* If yes, is it diagnosed pre pregnancy or during pregnancy ?

☐ Pre pregnancy ☐ Antenatally

** Please state the deranged OGTT result and time of gestation :

- OGTT result : fasting :__________/ 2 hours PP: ________

- Gestation of OGTT taken : at ______weeks

2- Any history of previous infant affected with GBS infection : * Yes / No

3- Any history of Sexually transmitted diseases (STD): * Yes / No

4- Any History of premature delivery (less than 37weeks) in previous pregnancy: * Yes / No

5- Any history of pervaginal bleeding throughout this pregnancy: * Yes / No

6- History of abnormal cervical screening result : * Yes / No

- If yes : select the abnormal result related : **

☐HPV positive / ☐ASCUS / ☐CIN 1 / ☐ CIN 2 / ☐CIN 3 / ☐others…………..

7- Any history of vaginal fungal infection (candidiasis) during this pregnancy : * Yes / No

8- Any constipation during this pregnancy since 3 months ago? : * Yes / No

- if **yes** : please fill in the **<u>ROME IV Questionnaire: Functional Constipation</u>** according to Bristol stool chart below:

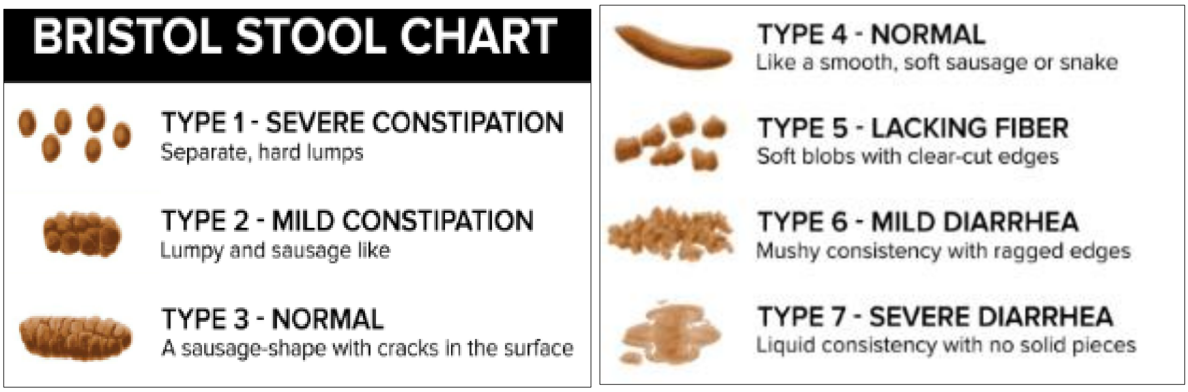

<u>8.1 : ROME IV Questionnaire: Functional Constipation</u>

1. In the last 3 months, how often did you have **hard or lumpy stools** that looked like Type 1 or 2 in the Bristol stool form scale? (Percent of all bowel movements)

0%<br>(never)	10%	20%	30%	40%	50%	60%	70%	80%	90%	100%<br>(always)

2. Did you have hard or lumpy stools (like Type 1 or 2) when you were not taking drugs for diarrhea?

No or rarely	Yes

3. In the last 3 months, how often did you have **fewer than three bowel movements a week** without taking a laxative medication or enema? (Percent of weeks)

0%<br>(never)	10%	20%	30%	40%	50%	60%	70%	80%	90%	100%<br>(always)

4. In the last 3 months, how often did you **strain during bowel movements**? (Percent of bowel movements)

0%<br>(never)	10%	20%	30%	40%	50%	60%	70%	80%	90%	100%<br>(always)

5. In the last 3 months, how often did you have a **feeling of incomplete emptying** after bowel movements? (Percent of bowel movements)

0%<br>(never)	10%	20%	30%	40%	50%	60%	70%	80%	90%	100%<br>(always)

6. In the last 3 months, how often did you have a **sensation that the stool could not be passed** (was blocked) when having a bowel movement? (Percent of bowel movements)

0% (never)	10%	20%	30%	40%	50%	60%	70%	80%	90%	100% (always)

7. In the last 3 months, how often did you **press on or around your bottom, or remove stool with your fingers**, in order to have a bowel movement? (Percent of bowel movements)

0% (never)	10%	20%	30%	40%	50%	60%	70%	80%	90%	100% (always)

8. Did any of the symptoms of constipation listed in questions 49–55 above begin more than 6 months ago?

No or rarely	Yes

9. In the last 3 months, how often did you have mushy or watery stools that looked like Type 6 or 7 in the Bristol stool form scale when you were not using drugs or other treatment for constipation? (Percent of all bowel movements)

0%	10%	20%	30%	40%	50%	60%	70%	80%	90%	100% (always)

10. Which of the following has been the most bothersome symptom for you in the last 3 months?

Abdominal pain	
Watery or mushy stools, or having many bowel movements in a day	
Hard stools or going several days without having a bowel movement	
Bloating or your belly looking unusually large	
None of the above, but another symptom (Please write that symptom below:)	

** RESULT FOR ANOVAGINAL SWAB FOR GBS : ☐POSITIVE ☐NEGATIVE

(** Result to be traced by Primary Investigator)
